# The effects of intravascular photobiomodulation on sleep disturbance caused by Guillain-Barré syndrome after Astrazeneca vaccine inoculation: Case report and literature review

**DOI:** 10.1097/MD.0000000000028758

**Published:** 2022-02-11

**Authors:** Yuan-Ling Chang, Shin-Tsu Chang

**Affiliations:** a Department of Medical Education and Research, Kaohsiung Veterans General Hospital, Kaohsiung, Taiwan; b Department of Anesthesiology, Kaohsiung Armed Forces General Hospital, No.2, Zhongzheng 1st Rd., Lingya Dist., Kaohsiung, Taiwan; c Department of Physical Medicine and Rehabilitation, Tri-Service General Hospital, School of Medicine, National Defense Medical Center, Taipei, Taiwan; d Department of Physical Medicine and Rehabilitation, Kaohsiung Veterans General Hospital, Kaohsiung, Taiwan.

**Keywords:** Covid-19, Guillain-Barré syndrome, Irradiation of blood, Oxford–AstraZeneca vaccine, pain modulation

## Abstract

**Rationale:**

Sleep disturbance is commonly noted after Guillain-Barré syndrome (GBS) and is often caused by persistent discomfort after disease survival. Intravascular laser irradiation of blood (ILIB) has been shown to be effective in pain modulation owing to the influence of nociceptive signals in the peripheral nervous system. We investigated the application of ILIB on post-Oxford –AstraZeneca vaccination GBS and evaluated its effect on sleep quality.

**Patient concerns:**

A 48-year-old woman was subsequently diagnosed with GBS after Oxford–AstraZeneca vaccination. The patient was discharged after a 5-day course of intravenous immunoglobulin administration. However, 1 week after discharge, the previously relieved symptoms flared with accompanying sleep disturbance.

**Diagnosis and interventions:**

The patient was diagnosed with post-vaccination GBS, and persistent pain and sleep disturbances persisted after disease survival. ILIB was performed.

**Outcomes:**

We used the Pittsburgh Sleep Quality Index before and after intravascular laser irradiation. There was a marked improvement in the sleep duration, efficiency, and overall sleep quality. The initial score was 12 out of 21 and the final score was 7 out of 21.

**Lessons:**

We found that ILIB was effective in pain modulation in post-vaccination GBS and significantly improved sleep quality.

## 1 Introduction

Covid-19, caused by the pathogen SARS-CoV-2, has become an ongoing pandemic that has spread worldwide since late 2019. The world has been racing to develop a vaccine to combat this devastating disease, and one of the vaccines developed is the Oxford–AstraZeneca vaccine. Interim results from ongoing trials in the UK and Brazil showed a promising vaccine efficacy of 70.4%.^[1]^ Although there is a clear benefit in vaccinating the general population, some side effects are now emerging as the number of vaccinated people has increased.

Guillain-Barré syndrome (GBS) is one such side effect, and despite being rare in occurrence, it often results in lingering pain and numbness even after disease survival. Sleep disturbance is commonly noted after GBS^[2]^; however, there is little discussion regarding vaccine-induced GBS. Intravascular laser irradiation of blood (ILIB) has been well documented to help with pain modulation due to the influence of nociceptive signals in the peripheral nervous system. We report the case of a middle-aged woman who survived vaccine-induced GBS but had persistent paresthesia and pain affecting her sleep quality. We administered ILIB therapy and hoped to reduce her discomfort and improve her sleep quality. We planned to use the Pittsburgh Sleep Quality Index (PSQI), which consists of 7 components–subjective sleep quality, sleep latency, sleep duration, sleep disturbance, use of sleep medication, and daytime dysfunction–to evaluate overall sleep quality improvement.

## 2 Case presentation

We report the case of a 48-year-old female of Japanese descent, with a past medical history of asthma and endometriosis under hormone replacement therapy, who experienced lower extremity numbness and paraesthesia 2 weeks after Oxford-AstraZeneca COVID-19 vaccine inoculation. The numbness started in her toes and then gradually extended to her ankles and upper extremities. She also developed progressive whole-body muscle soreness with paresthesia in both hands and feet. The patient's CoV-SARS polymerase chain reaction test via nasal swab was negative. The patient sought medical treatment 1 week after symptom onset, when her condition progressively worsened.

On physical examination, muscle power and deep tendon reflexes were normal, but mild imbalance was noted when asked to hop on 1 leg. Her pin-prick sensation was impaired from the bilateral soles to the ankles and hands (dorsal-side more pronounced). Magnetic resonance imaging (MRI) of the whole spine excluded herniated intervertebral discs and/or spinal stenosis. She was admitted and a lumbar puncture was performed. Cerebrospinal fluid analysis revealed albumin-cytological dissociation, while blood tests were essentially normal. Thus, GBS was suspected, and a 5-day course of intravenous immunoglobulin (IVIG) was administered. The patient was discharged and received outpatient rehabilitation and acupuncture.

However, 1 week after discharge, the previously relieved symptoms flared up again, this time with worsened prickling pain accompanied by numbness in her hands and feet. Gabapentin and prednisolone were prescribed as well as acupuncture therapy; however, the symptoms did not improve. However, the patient's symptoms did not improve. The patient stated that the pain was cyclical (about 2–3 hours a cycle) and persisted throughout the day, but was worse at night, waking her up 2 to 3 times a night, so she had to use Xanax as a sleeping aid approximately 2 to 3 times a week. The patient stated that there was no history of sleep disturbance, and that there were no other environmental factors responsible for sleep disturbance. There was also facial numbness and temporomandibular joint pain on mastication, with difficulty in tongue extension and swallowing. For the above reasons, the patient visited the NEURO outpatient department for help. Electromyography of the upper extremities revealed sensory-motor polyradiculoneuropathy, with severe demyelination and mild axonal degeneration, which is a typical finding after recent GBS.

**Figure 1:**
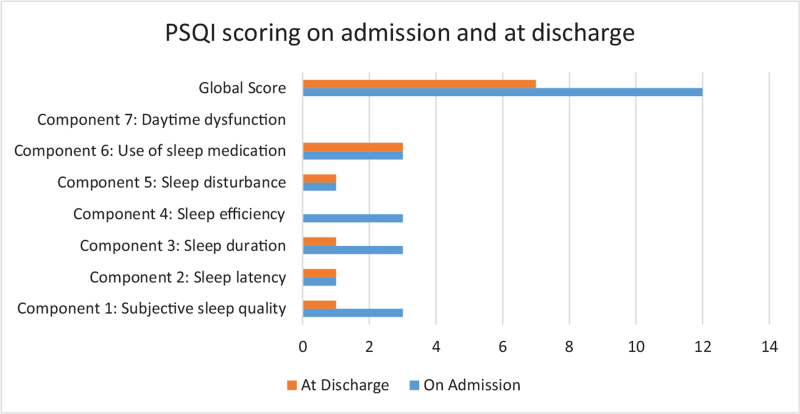
Pittsburgh Sleep Quality Index scoring on admission and at discharge.

Under NEURO specialist advice, she visited our rehabilitation department for further survey and inpatient rehabilitation. No sedatives, SSRIs, or central acting drugs were administered. However, gabapentin and xanax usage from prior to admission continued. The PSQI on admission revealed a score of 12 out of 21, which we used as baseline data (Fig. 1). No evidence of stroke or ischemic changes was seen on brain magnetic resonance imaging. After admission, ILIB was administered. An intravenous laser needle was inserted at the back of the hand, after cleaning the patient's skin with an alcohol wipe, and 632.8 nm infrared radiation was administered. The standard course of ILIB is composes of 10 sessions, each for 1 hour. The PSQI was re-tested on the day of discharge, after 5 days, and 5 courses of ILIB, which showed marked improvement, with a score of 7 out of 21 (Fig. 1). The remaining ILIB treatment courses were continued in our outpatient setting. Informed written consent was obtained from the patients in both English and Mandarin forms.

## 3 Discussion

GBS affects the peripheral nerves and nerve roots, presenting as ascending paralysis. It is usually preceded by certain infections (for example, such as *C. jejuni,* cytomegalovirus, and even COVID-19 ^[3]^), but has also been linked to other types of immune stimulation, such as vaccinations. Of the Covid-19 vaccines, GBS more often arises in Oxford–AstraZeneca and Janssen vaccines.^[4]^ A search of the major databases (PubMed, Cochrane, and Airiti) yielded 6 publications and ahead-of-prints^[5–10]^ on cases of GBS after Oxford–AstraZeneca vaccination, which we have summarized in Table 1.

**Table 1:** Summarization of post-AstraZeneca vaccination Guillain-Barre syndrome cases.

Names of Authors	Country/region	Case no.	Age/ ethnicity	1st/ 2nd dose	Time of Sx onset	Tx
^[5]^ Patel SU, et al, 2021	England	1 case	37 y/o male, Caucasian	1st	14 d	5 d of IVIG
^[6]^ Maramattom BV, et al, 2021	Kerala, India	7 cases	All of Indian descent: 1) 43 y/o female 2) 67 y/o female 3) 53 y/o female 4) 68 y/o female 5) 70 y/o male 6) 69 y/o female 7) 69 y/o female	1st	1) 10 d 2) 14 d 3) 12 d 4) 14 d 5) 11 d 6) 12 d 7) 13 d	1) IVIG + mechanical ventilation 2) IVIG+ mechanical ventilation+ plasmapheresis 3) IVIG + mechanical ventilation 4) IVIG + mechanical ventilation 5) IVIG+ mechanical ventilation 6) IVIG + plasmapheresis 7) IVIG + mechanical ventilation
^[7]^ Allen CM, et al, 2021	England	4 cases	1) 54 y/o male, Caucasian 2) 20 y/o male, British Iranian descent 3) 57 y/o male, Caucasian 4) 55 y/o male, Caucasian	1st	1) 16 d 2) 26 d 3) 21 d 4) 29 days	1) Oral prednisolone 2) Oral prednisolone 3) IVIG 4) No treatment needed
^[8]^ Hasan T, et al, 2021	England	1 case	62 y/o female, ethnicity unknown	1st	11 d	IVIG + mechanical ventilation + intravenous antibiotics (for secondary sepsis due to aspiration pneumonia)
^[9]^ Nasuelli NA, et al, 2021	Italy	1 case	59 y/o male, Caucasian	1st	10 d	IVIG
^[10]^ McKean N, et al, 2021	Malta	1 case	48 y/o male, presumed Maltese	1st	10 d	IVIG + oral prednisolone
Our case	Kaohsiung, Taiwan	1 case	48 y/o female, Japanese (East-Asian)	1st	14 d	IVIG

^∗^ Intravenous immunoglobulin (IVIG).

IVIG and plasma exchange have been proven to be effective treatments for GBS. Our patient had undergone a 5-day course of IVIG; however, residual pain, and paresthesia persisted. Residual complaints, such as pain, fatigue, and unsteady gait, frequently occur after patients survive GBS and can be attributed, in part, to persistent axonal loss. While the persistent discomfort in our patient was not life-threatening, it severely affected her quality of life. One of the most bothersome aspects of her persistent symptoms was the pain that wakes up at night, disrupting her sleep, and shortening her sleep duration.

ILIB is also known as photobiomodulation, and as the name suggests, uses “light” (also known as radiation) to modulate our bio-physiology, which has been used in stroke^[11]^ and CO intoxication.^[12]^ Many cellular molecules absorb various wavelengths of light and are effective in modulating nociceptive signals in the peripheral nervous system, which in turn translate to the central control of pain pathways.^[13]^

The PSQI was used to objectively assess sleep quality in our patient. Our patient's initial score on admission was 12 out of 21 and the final score on discharge was 7 out of 21 (the scoring of each component is summarized in Figure 1). There was a marked improvement in sleep duration, efficiency, and overall sleep quality, which we believe was due to the ILIB therapy.

## 4 Conclusion

Vaccination remains one of the most effective ways to combat the Covid-19 pandemic, however, we are now seeing some side effects in selected individuals. GBS, while rare, often causes lingering discomfort, even after survival. In many cases, the sleep quality is severely affected. Through the demonstration in our case, we believe that ILIB plays a pivotal role in patient recovery. We believe that there is a massive potential for the role of ILIB in post-vaccination GBS pain control.

## Author contributions

**Supervision:** Shin-Tsu Chang.

**Writing – original draft:** Yuan-Ling Chang.

## References

[1] Knoll MD Wonodi C. Oxford-AstraZeneca COVID-19 vaccine efficacy. Lancet 2021;397:72–4. 33306990 10.1016/S0140-6736(20)32623-4 PMC7832220

[2] Karkare K Sinha S Taly AB Rao S. Prevalence and profile of sleep disturbances in Guillain-Barre syndrome: a prospective questionnaire-based study during 10 days of hospitalization. Acta Neurol Scand 2013;127:116–23. 22642612 10.1111/j.1600-0404.2012.01688.x

[3] Palaiodimou L Stefanou MI Katsanos AH. Prevalence, clinical characteristics and outcomes of Guillain-Barré syndrome spectrum associated with COVID-19: a systematic review and meta-analysis. Eur J Neurol 2021;28:3517–29. 33837630 10.1111/ene.14860 PMC8250909

[4] Dyer O. Covid-19: regulators warn that rare Guillain-Barré cases may link to J&J and AstraZeneca vaccines. BMJ 2021;374:n1786. 34261628 10.1136/bmj.n1786

[5] Patel SU Khurram R Lakhani A Quirk B. Guillain-Barre syndrome following the first dose of the chimpanzee adenovirus-vectored COVID-19 vaccine, ChAdOx1. BMJ Case Rep 2021;14:e242956. 10.1136/bcr-2021-242956 PMC8070856 33888484

[6] Maramattom BV Krishnan P Paul R Padmanabhan S Cherukudal Vishnu Nampoothiri S. Guillain-Barré syndrome following ChAdOx1-S/nCoV-19 vaccine. Ann Neurol 2021;90:312–4. 34114256 10.1002/ana.26143

[7] Allen CM Ramsamy S Tarr AW. Guillain-Barré syndrome variant occurring after SARS-CoV-2 vaccination. Ann Neurol 2021;90:315–8. 34114269 10.1002/ana.26144

[8] Hasan T Khan M Khan F Hamza G. Case of Guillain-Barré syndrome following COVID-19 vaccine. BMJ Case Rep 2021;14:e243629. 10.1136/bcr-2021-243629 PMC8245439 34187803

[9] Nasuelli NA De Marchi F Cecchin M. A case of acute demyelinating polyradiculoneuropathy with bilateral facial palsy after ChAdOx1 nCoV-19 vaccine. Neurol Sci 2021;42:4747–9. 34272622 10.1007/s10072-021-05467-w PMC8285283

[10] McKean N Chircop C. Guillain-Barré syndrome after COVID-19 vaccination. BMJ Case Rep 2021;14:e244125. 10.1136/bcr-2021-244125 PMC8327820 34330729

[11] Liu CC Hsu CS He HC Cheng YY Chang ST. Effects of intravascular laser phototherapy on delayed neurological sequelae after carbon monoxide intoxication as evaluated by brain perfusion imaging: a case report and review of the literature. World J Clin Cases 2021;9:3048–55. 33969090 10.12998/wjcc.v9.i13.3048 PMC8080739

[12] Yang WH Lin SP Chang ST. Case report: rapid improvement of crossed cerebellar diaschisis after intravascular laser irradiation of blood in a case of stroke. Medicine (Baltimore) 2017;96:e5646. 28079797 10.1097/MD.0000000000005646 PMC5266159

[13] Chow RT Armati PJ. Photobiomodulation: implications for anesthesia and pain relief. Photomed Laser Surg 2016;34:599–609. 27419354 10.1089/pho.2015.4048

